# Investigation into the application of remimazolamin conjunction with low-dose propofolfor pediatricfiberoptic bronchoscopy

**DOI:** 10.1038/s41598-024-62181-1

**Published:** 2024-05-22

**Authors:** Wenjing Chen, Wenjuan Bao, Jing Shi, Lei Shi, Jianli Cui

**Affiliations:** Department of Anesthesiology, Hebei Children’s Hospital, No.133 Jianhua South Street, Shijiazhuang, 050031 Hebei China

**Keywords:** Remimazolam, Low-dose propofol, Fiberoptic bronchoscopy in children, Application analysis, Drug discovery, Medical research

## Abstract

This study delves into the effectiveness of combining remimazolam with low-dose propofol in pediatric fiberoptic bronchoscopy. Ninety children scheduled for fiberoptic bronchoscopy in our hospital were enrolled as research participants. Based on the intraoperative anesthetic drug regimen, the children were divided into three groups: group R (remimazolam 0.2–0.4 mg/kg), group P (propofol 1–3 mg/kg), and group RP (remimazolam0.2 mg/kg, propofol 0.5 mg/kg). Immediately post-anesthesia, group P exhibited lower blood pressure and heart rate (HR) compared to both group R and group RP (*P* < 0.05). As bronchoscope approached the glottis and epiglottis, group P continued to display lower blood pressure and HR compared to group R and group RP (*P* < 0.05). During lavage, group P maintained lower blood pressure and HR compared to both the R and RP groups (*P* < 0.05). Immediately post-anesthesia, group P demonstrated lower SpO_2_ compared to the R and RP groups (*P* < 0.05).During lavage, group P maintained lower SpO_2_ than group R and group RP (*P* < 0.05). In comparison with group R and group PR, group P showed shortened induction and recovery times (*P* < 0.05). The one-time entry success rate into the microscope was higher in group R than in group P, with the RP group showing an intermediate decreased (*P* < 0.05). Moreover, the cough score in R group was higher than in the P and RP groups (*P* < 0.05). Furthermore, the satisfaction rates of the RP group exceeded those of the R and P groups (*P* < 0.05). Remimazolam combined with low-dose propofol effectively balances the strengths and weaknesses of remimazolam and propofol, ensuring more stable hemodynamics, a lower incidence of adverse reactions, and optimal surgical conditions in pediatric fiberoptic bronchoscopy.

## Introduction

Fiberoptic bronchoscopy, a commonly employed endoscopic procedure for diagnosing and treating respiratory diseases in children, demands meticulous consideration in selecting anesthesia due to its precision and safety requirements^1–3^. Given the distinctive characteristics of pediatric patients, marked by heightened physiological sensitivity and reduced tolerance, the choice of an appropriate anesthesia method is crucial to ensuring smooth examination progress and the safety of children^4,5^. In clinical settings, remimazolam and propofol stand out as commonly used anesthetic drugs. Remimazolam, a benzodiazepine drug, has gained widespread use in various medical procedures owing to its sedative and anti-anxiety effects^6–8^. Noteworthy advantages include good tolerance and minimal respiratory depression. However, its slow onset of action and prolonged duration may lead to extended induction and awakening times^9,10^. In contrast, propofol is renowned for its rapid onset and short duration of action. It facilitates swift induction and recovery from anesthesia, making it suitable for procedures requiring promptcompletion^11–13^. Yet, propofol can induce significant cardiovascular and respiratory depression, especially in pediatric patients^14^. Given the distinct strengths and limitations of remimazolam and propofol, this study aims to explore whether their combined use, specifically remimazolam with low-dose propofol, can provide an optimal anesthesia regimen. The purpose of this combination is to harness the advantages of both drugs while mitigating potential adverse reactions associated with individual use. Particularly in pediatric population, this approach holds promise for enhancing the safety and effectiveness of anesthesia. In comparison to the exclusive use of remimazolam or propofol, this study seeks to provide a more comprehensive foundation for clinical anesthesia selection, ensuring the safety and comfort of children undergoing fiberoptic bronchoscopy.

## Materials and methods

### General information

From July 2023 to December 2023, this study enrolled 90 children scheduled for fiberoptic bronchoscopy in our hospital. The age range of the children was 6–12 years, with an average age of 9.44 ± 3.51 years. Among them, 48 were male, and 42 were female. The children were categorized into three groups: group R (remimazolam0.2–0.4 mg/kg), group P (propofol1-3 mg/kg) and group RP (remimazolam0.2 mg/kg and propofol 0.5 mg/kg). Informed consent was obtained from the guardians of all recruited children at the time of inclusion in the study. The research received approval from our hospital’s Ethics Committee (202407-27), and all guardians of the children provided informed consent.

### Inclusion criteria

Children aged 6–12 years, regardless of gender; Normal heart and lung function without major cardiopulmonary diseases; Requirement for fiberoptic bronchoscopy; Imminent need for bronchoscopy; Written consent from parents or legal guardians.

### Exclusion criteria

Children with known allergic reactions to remimazolam, propofol, or similar drugs; Presence of significant heart or lung diseases or other conditions affecting cardiopulmonary function; Congenital anatomical abnormalities impacting the safety of examination or anesthesia; Patients with asthma; Recent major surgery or severe physical trauma; History of drug dependence or abuse, particularly narcotic drugs or sedatives; Children with severe cognitive or behavioral disorders that may interfere with the examination or affect drug response.

### Anesthesia protocol

The child observed a 6 h fasting period, with intramuscular administration of 0.01 mg/kg atropine half an hour before the operation to facilitate venous access. Upon entering the operating room, non-invasive blood pressure, blood oxygen saturation, electrocardiogram, and respiratory rate were monitored. Bilateral nasal drops of and 2% lidocaine (0.5 ml on each side) were administered for topical anesthesia. Prior to induction, oxygen inhalation (4 L/min) was provided through a nasal catheter. All groups received a standard initial dose of sufentanil (0.05 μg/kg), followed by group R: 0.2–0.4 mg/kg of remimazolam, group P: 1–3 mg/kg of propofol, and group RP: 0.2 mg/kg of remimazolam and 0.5 mg/kg of propofol. The injection duration exceeded 60 s, and fiberoptic bronchoscopy commenced when the eyelash reflex disappeared, and consciousness was lost. Upon reaching the glottis during the fiberoptic bronchoscope, 1 ml of 2% lidocaine was injected into the trachea, and an additional 1 ml was injected when the fiberoptic bronchoscope reached the carina, with continued placement until completion of the examination. In cases of frequent body movements or a choking score exceeding 3 points, group Preceived an additional 1 mg/kg of propofol, while group RP and group R received 0.1 mg/kg of remimazolam. Children in each group underwent observation of the trachea, carina, and bronchi opening to assess the extent and progression of lung lesions. Alveolar lavage was administered to all children, and localized drugs were utilized to clear secretions or foreign bodies from each lung segment. Post-operation, samples were collected for inspection. In the event of hypoxemia (SpO_2_ less than 90), laryngeal spasm, or arrhythmia during the examination, the bronchoscope was withdrawn, and oxygen was administered via mask, followed by appropriate symptom management. If symptoms persisted, endotracheal intubation was performed to maintain oxygenation. Subsequent to the examination, the child was transferred to the post-anesthesia monitoring and treatment room and relocated to the ward when the improved Alderete score was ≥ 9.

### Observation indicators

The vital signs of children was monitored at various stages, including before anesthesia, immediately after anesthesia, at the glottis, epiglottis, during lavage, and after the operation. Parameter recorded included blood pressure (systolic and diastolic), oxygen saturation (SpO_2_), and heart rate (HR). Perioperative indicators such as induction time, examination time, awakening time, leaving the room time, and the frequency of drug additions were recorded. The success rate of one-time endoscope entry and the choking score for each child were compared among the groups. The success rate of one-time examination the number of cases successfully intubated through fiberoptic bronchoscopy once/the total number of cases × 100.00%.The choking score was categorized as follows: 1 point for no choking, 2 points for 1 or 2 instances of mild coughing, 3 points for 3 or 4 instances of moderate coughing, and 4 points for 5 or more instances of severe coughing. Adverse reactions during the perioperative period, such as hypoxemia, hypotension, nausea, vomiting, hiccups, and injection pain, were recorded. The mini-mental state examination (MMSE) scoring tool was used to evaluate the cognitive function of children before and after the operation. MMSE includes a series of short tests and questions evaluating memory, attention, calculation ability, language, and visual space skills, with a total score of 30.The higher the score, the more indicative of normal cognitive function. A satisfaction questionnaire was issued post-operation to gauge children’s satisfaction with the sedation scheme.

### Statistical analysis

Statistical analyses were performed using GraphPad Prism 5. Data were presented as mean ± SD. Students’ *t*-test (for comparison between two groups) or one-way analysis of variance (ANOVA, for comparisons among three or more groups) was applied for statistical comparisons, followed by Bonferroni post hoc testing. A significance level of *P* < 0.05 was considered statistically significant.

### Ethics approval and consent to participate

The current study was conducted in accordance with the Helsinki Declaration of the World Medical Association and approved by the Ethics Committee of Hebei Children’s Hospital. Informed consent was obtained from all the study subjects before enrollment.

## Results

### General data analysis of children

The distribution of male to female ratios in group R was 14:16, with an average age of 10.04 ± 2.77 years, an average height of 143.67 ± 4.26 cm, an average weight of 36.58 ± 3.44 kg, and an average BMI of 16.56 ± 3.25 kg/m^2^. In group P, the male-to-female ratio was 18:12, with an average age of 9.14 ± 3.51 years, an average height of 147.38 ± 5.19 cm, an average weight of 35.16 ± 4.56 kg, and an average BMI of 15.24 ± 3.08 kg/m^2^. The ratio of males to females in the RP group was 17:13, with an average age of 9.75 ± 3.06 years, an average height of 144.26 ± 4.27 cm, an average weight of 33.72 ± 4.39 kg, and an average BMI of 15.64 ± 3.22 kg/m^2^. No significant differences were observed in the general data among the three groups (*P* > 0.05). (Table 1).

**Table 1:** General data analysis of children.

Parameter	Group R (n = 30)	Group P (n = 30)	Group RP (n = 30))	Variance ratio	*P* value
Gender (male: female)	14: 16	18: 12	17:13	4.017	0.604
Age (years)	10.04 ± 2.77	9.14 ± 3.51	9.75 ± 3.06	3.226	0.221
Height	143.67 ± 4.26	147.38 ± 5.19	144.26 ± 4.27	5.371	0.376
Weight	36.58 ± 3.44	35.16 ± 4.56	33.72 ± 4.39	6.385	0.295
BMI(kg/m^2^)	16.56 ± 3.25	15.24 ± 3.08	15.64 ± 3.22	4.113	0.221

### Monitoring of vital signs during anesthesia

The vital signs of children in the three groups were monitored and compared through the anesthesia process. Prior to anesthesia, no significant differences were seen in blood pressure (systolic and diastolic) and HR among the three groups (*P* > 0.05). Immediately after anesthesia, group P exhibited lower blood pressure (systolic and diastolic) and HR compared to group R and group RP (*P* < 0.05), with both groups maintaining relatively stable blood pressure and HR. Upon reaching the glottis, similar trends were observed, with group P displaying lower blood pressure (systolic and diastolic) and HR compared to group R and group RP (*P* < 0.05), and both maintained more stable blood pressure and HR. During the lavage phase, group P continued to exhibit lower blood pressure (systolic and diastolic) and HR compared to group R and group RP (*P* < 0.05), while group R and group RP demonstrated more stable blood pressure. Post-operation, the blood pressure (systolic and diastolic) and HR gradually returned to normal in all three groups, with no significant differences observed (*P* > 0.05).Notably, the recovery of blood pressure in group P was slower than in the other groups. Prior to anesthesia, there were no notable differences in SpO_2_ among the three groups (*P* > 0.05). Immediately after anesthesia, SpO_2_ in group P was lower than in group R and group RP (P < 0.05), with no significant difference between group R and group RP (*P* > 0.05). While SpO_2_remained relatively stable in group R and group RP, it continued to be lower in group P (*P* < 0.05). Similar trends were observed during lavage, with group P exhibiting lower SpO_2_ compared to group R and group RP (*P* < 0.05), while stability was maintained in group R and group RP. Post-operation, SpO_2_ gradually returned to normal in all groups, with no significant differences observed (*P* > 0.05). However, the recovery of SpO_2_ in group P was notably slower. (Table 2).

**Table 2:** Monitoring of vital signs of children during anesthesia.

Time point	Group R(n = 30)	Group P (n = 30)	Group RP (n = 30)	Variance ratio	*P* value
Before anesthesia					
SBP(mmHg)	112.45 ± 6.35	109.66 ± 5.37	110.26 ± 6.41	4.118	0.524
DBP(mmHg)	73.22 ± 4.25	75.18 ± 5.48	72.59 ± 6.31		
HR (times/min)	95.36 ± 5.22	93.17 ± 6.38	97.41 ± 5.51		
SpO_2_ (%)	98.45 ± 1.66	98.68 ± 1.83	99.41 ± 1.02		
Immediate anesthesia					
SBP(mmHg)	107.35 ± 7.38	86.43 ± 6.54	102.66 ± 6.41	11.403	0.001
DBP(mmHg)	75.51 ± 5.44	54.29 ± 4.31	68.38 ± 6.28		
HR (times/min)	117.35 ± 7.33	87.52 ± 6.21	108.54 ± 6.39		
SpO_2_ (%)	98.35 ± 2.41	94.44 ± 1.65	97.31 ± 1.74		
Glottis entry					
SBP(mmHg)	114.21 ± 5.49	89.37 ± 4.18	105.35 ± 6.37	17.504	0.001
DBP(mmHg)	79.21 ± 5.64	57.19 ± 4.34	73.64 ± 4.45		
HR (times/min)	125.73 ± 6.59	90.44 ± 5.37	114.67 ± 5.39		
SpO_2_ (%)	95.32 ± 1.14	91.64 ± 1.58	94.22 ± 1.08		
Epiglottis entry					
SBP(mmHg)	109.57 ± 10.66	83.65 ± 9.71	94.36 ± 8.65	14.622	0.001
DBP(mmHg)	83.38 ± 8.29	55.24 ± 7.33	78.52 ± 8.26		
HR (times /min)	130.44 ± 4.27	96.33 ± 5.17	121.54 ± 3.88		
SpO_2_ (%)	94.62 ± 2.33	90.67 ± 1.85	92.54 ± 1.27		
During lavage					
SBP(mmHg)	105.26 ± 6.21	87.11 ± 5.72	100.57 ± 4.22	9.463	0.002
DBP(mmHg)	86.11 ± 3.28	57.49 ± 4.55	80.52 ± 4.47		
HR (times/min)	126.51 ± 6.33	98.42 ± 5.40	122.75 ± 4.95		
SpO_2_ (%)	95.26 ± 2.16	93.55 ± 1.86	94.25 ± 1.66		
Operation completion					
SBP(mmHg)	115.33 ± 5.18	110.63 ± 4.96	107.36 ± 5.38	3.015	0.227
DBP(mmHg)	75.62 ± 4.31	77.35 ± 5.24	75.51 ± 4.31		
HR (times /min)	105.36 ± 6.32	101.73 ± 5.42	103.56 ± 6.19		
SpO_2_ (%)	98.61 ± 1.65	98.75 ± 1.35	99.12 ± 1.22		

### Operation time and number of additional drugs

Group P exhibited a reduction in both induction time and recovery time compared to the R group and PR group (*P* < 0.05). However, this improvement was accompanied by an increased frequency of drug administration in the P group (*P* < 0.05). In contrast, the R group experienced longer induction and recovery times but required fewer additional drugs (*P* < 0.05). (Table 3).

**Table 3:** Operation time and times of adding drugs.

Index	Group R (n = 30)	Group P (n = 30)	Group RP (n = 30)	Variance ratio	*P* value
Induction time (minutes)	4.36 ± 1.22	1.97 ± 0.75	2.65 ± 1.04	14.378	0.001
Operation time (minutes)	20.64 ± 4.38	19.26 ± 5.11	21.36 ± 4.26	5.208	0.278
Awakening time (minutes)	24.64 ± 4.37	13.25 ± 2.48	16.33 ± 2.76	12.485	0.001
Time out of the room (minutes)	57.38 ± 8.45	38.65 ± 5.22	45.34 ± 6.28	9.203	0.00
Number of additional drugs	1.46 ± 0.56	2.33 ± 0.52	1.78 ± 0.61	15.447	0.001

### Comparison of success rate and choking score of one-time lens entry

When evaluating the success rate and choking score of a one-time lens entry children in group R demonstrated a lower success rate compared to group P and group RP (*P* < 0.05). Additionally, and the choking score in group R was higher than that in group P and group RP (*P* < 0.05), with no significant difference observed between group P and group RP (*P* > 0.05). (Table 4).

**Table 4:** Success rate and choking score of one-time lens entry.

Index	Group R(n = 30)	Group P (n = 30)	Group RP (n = 30)	Variance ratio	*P* value
Success rate of one-time lens entry	20(66.66%)	26(86.66%)	24(80.00%)	16.215	0.000
Choking score	3.45 ± 0.37	1.65 ± 0.20	1.74 ± 0.26	11.564	0.001

### Adverse anesthesia reactions

**Figure 1:**
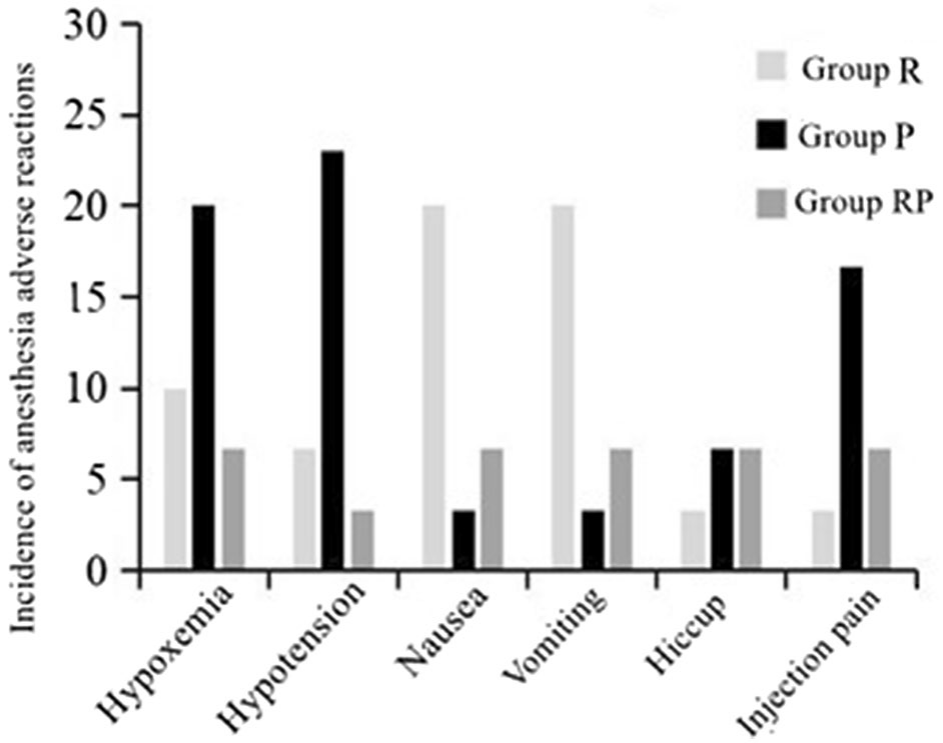
Incidence of adverse anesthesia reactions.

In the analysis of adverse reactions, the incidence of hypotension, hypoxemia, and injection pain in group P was higher than that in group R and group RP (*P* < 0.05). However, there was no significant difference between group R and group RP (*P* > 0.05). Moreover, the incidence of nausea and vomiting was higher in R group compared to the P and RP groups (*P* < 0.05), with no significant difference between the P group and RP groups (*P* > 0.05). The occurrence of hiccups did not differ significantly among the three groups (*P* > 0.05). The RP group effectively combined the advantages of both drugs, resulting in a reduction in side effects. (Fig. 1, Table 5).

**Table 5:** Adverse anesthesia reactions.

Adverse reactions (%)	Group R(n = 30)	Group P (n = 30)	Group RP (n = 30)	Variance ratio	*P* value
Hypoxemia	3(10.00%)	6(20.00%)	2(6.67%)	10.356	0.003
Hypotension	2(6.67%)	7(23.33%)	2(6.67%)	15.419	0.001
Nausea	6(20.00%)	1(3.33%)	1(3.33%)	10.552	0.002
Vomiting	6(20.00%)	1(3.33%)	2(6.67%)	9.042	0.002
Hiccup	1(3.33%)	2(6.67%)	1(6.67%)	3.215	0.573
Injection pain	1(3.33%)	5(16.66%)	1(6.67%)	12.556	0.001

### Comparison of cognitive function (MMSE) scores

**Figure 2:**
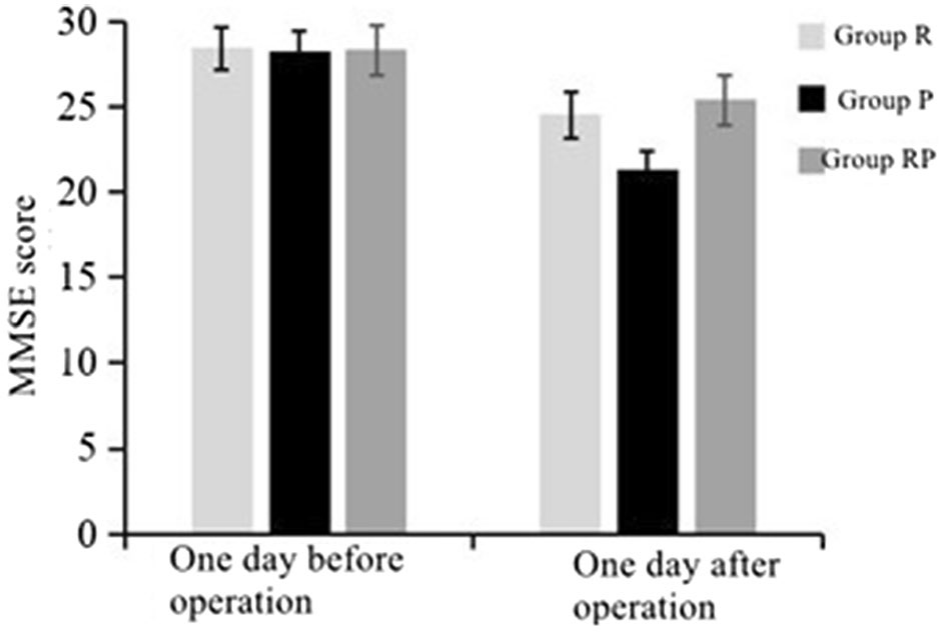
Comparison of cognitive function (MMSE) scores.

When assessing cognitive function using MMSE scoring system, there was no significant difference in MMSE scores among the three groups on the day before the operation (*P* > 0.05). However, on the day after operation, the MMSE scores of group P were lower than those of group R and group RP (*P* < 0.05), with no significant difference between group R and group RP (*P* > 0.05). (Fig. 2, Table 6).

**Table 6:** Comparison of cognitive function (MMSE) scores.

Index	Group R(n = 30)	Group P (n = 30)	Group RP (n = 30)	Variance ratio	*P* value
One day before operation	28.45 ± 1.22	28.26 ± 1.37	28.36 ± 1.44	2.106	0.447
One day after operation	24.57 ± 1.25	21.35 ± 1.04	25.44 ± 1.21	18.527	0.001

### Satisfaction questionnaire

In terms of satisfaction with the sedation scheme, both the very satisfactory rate and total satisfactory rate were higher in the RP group compared to the R group and P groups (*P* < 0.05) (Table 7).

**Table 7:** Satisfaction questionnaire.

Satisfaction (%)	Group R(n = 30)	Group P (n = 30)	Group RP (n = 30)	Variance ratio	*P* value
Very satisfied	22(73.33%)	19(63.33%)	28(93.33%)	12.538	0.001
Satisfied	5(16.66%)	5(16.66%)	1(3.33%)	9.114	0.005
Not satisfied	4(10.01%)	6(20.01%)	1(3.33%)	15.655	0.034
Total satisfaction rate	26(86.67%)	24(80.00%)	29(96.67%)	13.417	0.001

## Discussion

This study delves into the anesthetic effects of remimazolam, propofol, and their combination in children undergoing fiberoptic bronchoscopy, with a particular focus on key vital signs such as blood pressure, HR, and SpO_2_. The findings unveil significant differences among the three anesthesia schemes in these vital aspects, offering crucial insights for selecting the most appropriate anesthesia method in clinical settings. The research indicates that children anesthetized with propofol (group P) exhibited notably lower blood pressure and HR immediately after anesthesia, at the time of reaching glottis and epiglottis, and during lavage compared to those in children administered only with remimazolam (group R) or the combination of remimazolam and propofol (group RP). This observation suggests the robust cardiovascular inhibitory effect of propofol, especially in the decline of HR and blood pressure. Such pronounced cardiovascular inhibition might present challenges to the stability of children, particularly in surgeries with stringent requirements for cardiovascular function. Conversely, both the R group and RP group demonstrated better in maintaining hemodynamic stability, possibly indicating the mild cardiovascular impact of remimazolam and its synergistic effect when combined with propofol^15,16^. These approaches exhibit favorable attributes in sustaining stable blood pressure and heart rate in children. Moreover, in terms of oxygen saturation, children in group P exhibited lower levels, potentially attributed to respiratory depression induced by propofol. This is of particular concern in pediatric anesthesia, given the heightened sensitivity of children to changes in oxygenation status. On the contrary, children in the R group and RP group maintained a more stable SpO_2_ level, indicating potential superiority in preserving respiratory function. Especially in the RP group, while side effects from single-drug use are minimized, a good oxygenation state is maintained, showing potential advantages in pediatric anesthesia.

The study also focused on two crucial indicators, operation-related time, and the number of additional drugs, to evaluate the application effect of various anesthesia protocols in children undergoing fiberoptic bronchoscopy. The results indicate that children administered with propofol group P) exhibit notable advantages in induction and awakening time. This can be attributed to the pharmacological characteristics of propofol, characterized by rapid onset and clearance^17,18^. The swift induction and recovery times associated with propofol present a significant advantage for procedures that require expeditious completion^19,20^. However, the rapid metabolism of propofol implies a potential need for more frequent drug additions during the operation to maintain the required depth and effectiveness of anesthesia. In contrast, the R group, solely administered with remimazolam, demonstrated longer induction and awakening times. This delay may stem from the slow onset and prolonged action duration of remimazolam, leading to an extended overall anesthesia period^21,22^. However, owing to its prolonged action, group R required fewer additional drugs during the operation. This indicates that remimazolam might be more suitable for situations requiring extended maintenance of anesthesia, particularly in situations where frequent intervention in anesthesia is undesired. These findings emphasize the importance of considering onset time, duration, and additional drug requirements comprehensively when selecting anesthesia protocols for children undergoing fiberoptic bronchoscopy.

In examining the application of remimazolam, propofol, and their combination in children undergoing fiberoptic bronchoscopy, we conducted a detailed analysis of adverse reaction incidence, cognitive function assessment, and patient satisfaction. The findings revealed a significantly higher incidence of hypotension, hypoxemia, and injection pain in children administered with propofol alone (group P) compared to those in children administered only with remimazolam (group R) or the combination of remimazolam and propofol (group RP). This discrepancy may be linked to the potent respiratory and cardiovascular inhibitory effects of propofol^23^. Conversely, group R exhibited a higher incidence of nausea and vomiting, likely attributable to the common side effects of remimazolam. The RP group demonstrated distinct advantages in adverse reaction incidence, indicating that the combined use of remimazolam and propofol can mitigate some of their individual side effects, providing a more balanced anesthetic effect. Regarding cognitive function, there was no significant difference in MMSE scores among all groups before operation. However, one day after the operation, the score in group P significantly decreased, possibly reflecting the short-term negative impact of propofol on cognitive function^24^. In contrast, the cognitive function of children in the R group and RP group exhibited better recovery one day after the operation, indicating that these anesthesia protocols have minimal influence on cognitive function. Finally, based on the satisfaction evaluation of children and their parents, the RP group scored higher than the R group and group P. This implies that the combined use of remimazolam and propofol presents significant advantages in improving patients’ comfort and satisfaction. This emphasizes the importance of considering drug side effects and their impact on the overall patient experience when selecting an anesthesia protocol for children undergoing fiberoptic bronchoscopy, aiming to achieve optimal clinical outcomes and patient satisfaction^25^.

The combined administration of remimazolam and propofol (RP group) emerges as a highly favorable and ideal anesthesia option for children undergoing fiberoptic bronchoscopy. This combination exhibits notable strengths in maintaining the stability of blood pressure and heart rate, mitigating the common cardiovascular inhibition issues associated with sole propofol use, such as marked blood pressure drops and heart rate changes. Simultaneously, the RP group demonstrates significantly lower incidences of hypoxemia and injection pain compared to group P using propofol alone, indicating that the combined approach can alleviate respiratory depression and injection discomfort associated with propofol. Furthermore, essential surgical efficiency metrics in the RP group, including the success rate of one-time lens entry and choking score, mirror those of propofol group. This suggests that the combination of remimazolam and propofol not only upholds optimal surgical conditions but also improves the procedural smoothness. In terms of children’s comfort, the RP group outperforms the R group, which solely utilized remimazolam, by reducing the incidence of nausea and vomiting, thereby minimizing postoperative discomfort for the children.

Remazolam is a new ultra-short-acting intravenous sedative hypnotic for benzodiazepines, which can significantly shorten the sedative onset and recovery time, and can be antagonized by flumazenil. Propofol is the most widely used anesthetic in rigid bronchoscopy, but there is no specific antagonist, and its elimination is mainly caused by cardiac displacement and liver metabolism. Remazolam combines the sedative effectiveness of propofol, the sedative safety of midazolam, and the qualities of metabolic pathways similar to remifentanil. Remazolam combined with propofol had the highest sedation success rate, the shortest sedation onset time and the shortest recovery time, and no adverse reactions were observed. Bronchoscopy is a kind of high-risk outpatient surgery. Proper sedation can reduce stress and play a certain role in airway protection. Remazolam combined with propofol is expected to provide a new choice for clinical anesthesia.

In a word, the outcomes of this study underscore the considerable advantages of employing remimazolam in combination with low-dose propofol for children undergoing fiberoptic bronchoscopy. This combination effectively balances the strengths and weaknesses of remimazolam and propofol, offering more stable hemodynamics, a lower incidence of adverse reactions, and favorable surgical conditions. Therefore, the combined use of remimazolam and low-dose propofol emerges as an ideal anesthetic choice for pediatric fiberoptic bronchoscopy.

## Data Availability

The datasets generated and analyzed during the current study are available from the corresponding author on reasonable request.

## Abbreviations

HR: Heart rate
MMSE: Mini-mental state examination
